# Using Health Check Data to Understand Risks for Dementia and Cognitive Impairment Among Torres Strait Islander and Aboriginal Peoples in Northern Queensland—A Data Linkage Study

**DOI:** 10.3389/fpubh.2022.782373

**Published:** 2022-02-16

**Authors:** Fintan Thompson, Sarah G. Russell, Linton R. Harriss, Adrian Esterman, Sean Taylor, Rachel Quigley, Edward Strivens, Robyn McDermott

**Affiliations:** ^1^College of Public Health, Medical and Veterinary Sciences, Australian Institute of Tropical Health and Medicine, James Cook University, Cairns, QLD, Australia; ^2^College of Medicine and Dentistry, James Cook University, Cairns, QLD, Australia; ^3^Queensland Health, Cairns and Hinterland Hospital and Health Service, Cairns, QLD, Australia; ^4^Clinical and Health Sciences, University of South Australia, Adelaide, SA, Australia; ^5^Top End Health Service, Northern Territory Government, Darwin, NT, Australia

**Keywords:** dementia, first nation, Indigenous, cognitive impairment, Australia, Aboriginal, Torres Strait Islander

## Abstract

**Objective:**

High rates of dementia are evident in First Nations populations, and modifiable risk factors may be contributing to this increased risk. This study aimed to use a longitudinal dataset to gain insights into the long-term risk and protective factors for dementia and cognitive impairment not dementia (CIND) in a Torres Strait Islander and Aboriginal population in Far North Queensland, Australia.

**Study Design and Setting:**

Probabilistic data linkage was used to combine baseline health check data obtained in 1998/2000 and 2006/2007 for 64 residents in remote communities with their results on a single dementia assessment 10–20 years later (2015–2018). The relationship between earlier measures and later CIND/dementia status was examined using generalized linear modeling with risk ratios (RRs). Due to the small sample size, bootstrapping was used to inform variable selection during multivariable modeling.

**Results:**

One third of participants (*n* = 21, 32.8%) were diagnosed with dementia (*n* = 6) or CIND (*n* = 15) at follow-up. Secondary school or further education (RR = 0.38, 95% CI 0.19–0.76, *p* = 0.006) and adequate levels of self-reported physical activity (RR = 0.26, 95% CI 0.13–0.52, *p* < 0.001) were repeatedly selected in bootstrapping and showed some evidence of protection against later CIND/dementia in final multivariate models, although these had moderate collinearity. Vascular risk measures showed inconclusive or unexpected associations with later CIND/dementia risk.

**Conclusions:**

The preliminary findings from this small study highlighted two potential protective factors for dementia that may be present in this population. A tentative risk profile for later CIND/dementia risk is suggested, although the small sample size limits the applicability of these findings.

## Introduction

Dementia is a major global public health concern, affecting an estimated 55 million people worldwide (1). Rates are projected to increase globally in the coming decades, albeit with variations at regional levels (2). While dementia incidence has plateaued in certain developed countries (3), in developing countries and disadvantaged groups, the rates have increased and are likely to remain elevated (4). First Nations populations experience elevated rates of dementia and cognitive impairment, with potentially modifiable risk factors likely contributing to this disparity (5).

Aboriginal and Torres Strait Islander peoples are the First Nations inhabitants of Australia and among oldest continuous populations in the world (6). Their diverse cultures value family, community, and connection to country, with rich kinship systems and rules that govern social interaction, law, education, and resource management (7). As a result of the devastating and enduring impact of European colonization, Aboriginal and Torres Strait Islander peoples experience greater exposure to life course risk factors for dementia compared with other Australians (e.g., intergenerational trauma, social disadvantage, and chronic disease). Correspondingly, this group also experiences rates of dementia 3–5 times higher compared with other Australians (8–10). Research on risk factors for dementia in Aboriginal and Torres Strait Islander peoples has been informative, although most studies are cross-sectional (5, 11, 12) or limited to 5-to-6-year follow-up periods (13, 14). As a result, there is currently sparse evidence on life course risk factors for dementia in these populations, which limits accurate identification of individuals most at risk of developing dementia in these communities and also identifying which protective factors are most important.

The Torres Strait and Northern Peninsula Area is a geographic region of Australia that stretches from the tip of Cape York in Queensland to islands within sight of the Papua New Guinean coast. The region includes 18 inhabited islands and five mainland communities. The majority of the population identifies as Torres Strait Islander, a culturally, historically, and linguistically diverse Australian First Nations population, traditionally of Melanesian descent. In 2016, ~9,000 people who identified as Torres Strait Islander and/or Aboriginal were estimated to be living in this region (15, 16). A recent cross-sectional study of 276 Torres Strait Islander and Aboriginal peoples from this region indicated this population had a threefold risk of dementia compared with other Australians (the Dementia Prevalence Study, 2015–2018) (11). The same project reported that cerebrovascular disease and chronic kidney disease were associated with dementia risk (17). Participants in this study were also diagnosed with cognitive impairment not dementia (CIND) (i.e., mild cognitive impairment) if they had cognitive decline beyond age expectations and were functionally intact. This diagnosis reflects a heightened risk of future dementia and is clinically important in terms of early identification and dementia prevention (18).

A subset of participants in the Dementia Prevalence Study also took part in a community health check, which was undertaken as part of a separate research project in the same geographic regions 10–20 years earlier (the Well Person's Health Check, WPHC, 1998/2000 and 2006/2007) (19). This occurrence provides an opportunity to combine information for individuals who participated in both research projects to create a longitudinal dataset.

The aim of the current study was to use a longitudinal dataset to gain insights into the long-term risk and protective factors for dementia and cognitive impairment among a Torres Strait Islander and Aboriginal population in Far North Queensland. The objectives were to (i) link data from the Dementia Prevalence Study and the WPHC for people who participated in both research projects, (ii) analyze this linked dataset to examine the relationship between risk and protective factors earlier in life with measures of cognitive assessment in later life, and (iii) develop a crude health profile, which would describe the health characteristics of an individual in this population who may be at later risk of dementia or CIND. The purpose of this was to communicate results back to the community and the local health service to inform service delivery.

## Materials and Methods

### Study Design and Population

This retrospective cohort study was based on datasets from two separate research projects. These were the WPHC (19) and the Dementia Prevalence Survey (11).

The WPHC was a community based screening program of 3,033 Aboriginal and Torres Strait Islander individuals aged 13 years and over living in 26 rural and remote communities in northern Queensland, Australia, between March 1998 and December 2000 (19). Non-Indigenous residents and children aged ≤ 12 years were excluded from the study. An additional WPHC was undertaken between 2005 and 2007 in a subset of communities, which involved the follow up of some participants and recruitment of new participants (20, 21).

The Dementia Prevalence Survey was a cross-sectional assessment of the prevalence of dementia among 276 Torres Strait Islander and Aboriginal peoples aged 45 years and over living in all the populated island and mainland communities in the Torres Strait and Northern Peninsula Area of Far North Queensland between 2015 and 2018 (11). Participants were screened with an adapted version of the Kimberley Indigenous Cognitive Assessment (KICA) tool and received a clinical assessment for dementia or CIND, as part of a comprehensive geriatric assessment.

The WPHC and the Dementia Prevalence Survey overlapped in seven communities in the Torres Strait and five communities of the Northern Peninsula Area. In the years between the studies, some participants had moved to a community that was included in the Dementia Prevalence Survey and not the original WPHC. As a result, the current study comprised participants from eight communities in the Torres Strait and five communities of the Northern Peninsula Area. Ethics approval to link the datasets from these two studies was granted on 07/12/2018 by the Far North Queensland Human Research Ethics Committee (HREC/18/QCH92-1262).

### Sample Size

A sample size calculation using the “Kelsey formula” for an independent cohort study (22) indicated 273 participants would be required [i.e., α = 0.05, β = 0.80, Prevalence not Exposed (P0) = 0.28, Prevalence Exposed (P1) = 0.44, Relative Risk (RR) = 1.6, Ratio of exposed to unexposed (r) = 1.00]. The parameters for this formula were based on a smaller pilot linkage of the data where albuminuria showed a 60% increase in later life CIND/Dementia risk (i.e., RR = 1.6).

### Data Linkage

**Figure 1:**
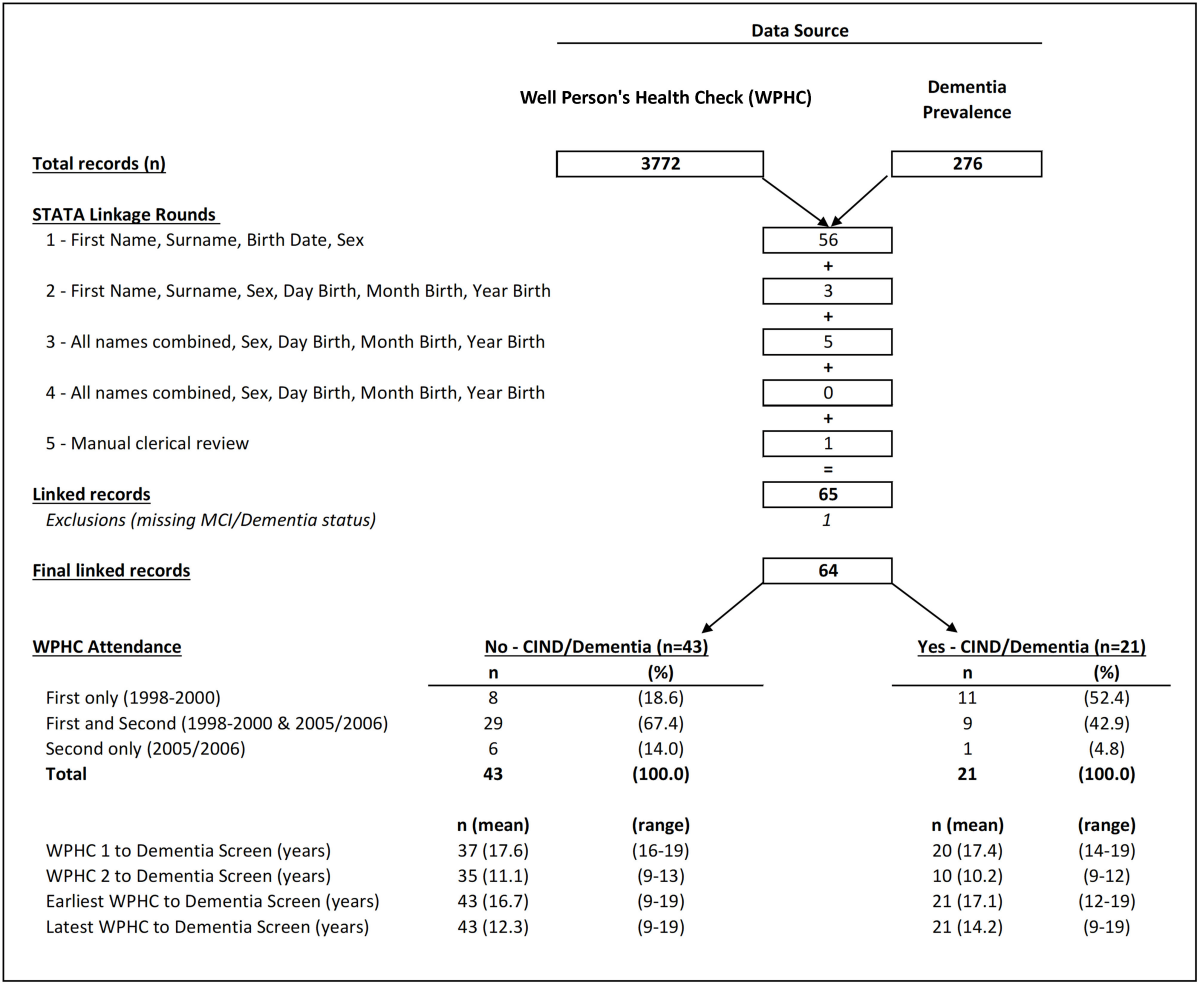
Flowchart showing steps involved in probabilistic linkage of datasets from the Well Person's Health Checks (WPHC) (1998/2000, 2005/2006) and the Dementia Prevalence Survey (2015–2018). CIND, Cognitive Impairment Not Dementia; WPHC1, First Well Person's Health Check (1998–2000); WPHC2, Second Well Person's Health Check (2005–2006); Earliest WPHC, Earliest recorded information from the first WPHC screen if the person attended, or the second screen if the person did not attend the first screen; Latest WPHC, Latest recorded information was from the second WPHC screen if the person attended, or the first screen if the person did not attend the second screen.

Probabilistic data linkage was undertaken over four stages by the primary author (FT) in Stata 15 (College Station, TX: StataCorp LLC) using the package “dtalink.” The weights and thresholds that defined “Exact Matches” and matches requiring review are described in Supplementary Figure 1. The Stata “calcweights” command determined the weighting for identifiers at each stage. At the end of each stage, successfully linked records were removed from each dataset, so the number of unlinked records in both datasets was reduced for the next stage. After four rounds of probabilistic linkage, a final review of both datasets was also undertaken. A final linked dataset of identifiers from both data sources was prepared and provided to a co-author for review (ST). A Unique Linkage Key was generated and used to combine the full WPHC and Dementia Prevalence Survey datasets into a final linked study dataset for analyses (Figure 1).

### Predictor Variables—Description

A detailed description of the WPHC methods has been published elsewhere (19). The WPHC dataset contained baseline information on waist circumference (centimeters, cm), weight (kilograms, kg), Body Mass Index (BMI), current alcohol consumption (Yes/No), and cigarette smoking (i.e., self-reported “currently smoked tobacco,” Yes/No). Food intake was measured *via* a 24-h recall method, which included the number of serves of fruit and vegetables. Physical activity was measured using a 7-day recall method. Adequate levels were ≥5 days in the previous 7 days where a participant undertook moderate physical activity for ≥20 min. Hypertension (HBP) was defined as systolic ≥140 mmHg and/or diastolic ≥90 mmHg, and/or current anti-hypertensive medication. Fasting venous blood samples were used to collect measures for triglycerides, total cholesterol, high density lipoprotein (HDL), low density lipoprotein (LDL), gamma-glutamyl transferase (GGT), and fasting glucose. Very Low-Density Lipoprotein (VLDL, mmol/l) was derived *post-hoc* from triglycerides. Diabetes was identified through self-report (confirmed through medical record check) or a baseline fasting glucose >7.8 mmol/l. Hyperglycemia (HBG) was defined as fasting glucose ≥5.5 mmol/l. First catch urine samples were self-collected and analyzed for albumin creatinine ratio (UACR). Albuminuria, which is an excess of the protein albumin in the urine and an early indicator of kidney damage and a surrogate marker systematic microvascular dysfunction (23), was defined as UACR ≥2.5 mg/mmol for males and ≥3.5 for females (24).

### Predictor Variables—Categorization

There was variation in screening attendance across the two WPHC time points. For example, some participants had first WPHC data only, others had second WPHC only, and some had data from both health checks (see Figure 1). There was also variation in the completeness of the baseline measures during each screening (see Supplementary Table 1). To ensure maximum completeness of baseline information and the greatest number of participants, this study used the “earliest” and “latest” WPHC measures available for each participant. The “earliest” available measure was data from the first WPHC screen, supplemented with data from the second screen for participants who had not attended the first one. The “latest” available measure was the reverse, that is, data from the second WPHC screen for participants who attended, supplemented with information from the first screen for participants who had not attended the second one. The main two analyses for this study were the relationship between 1) each participant's earliest WPHC measures and later dementia assessment and 2) their latest WPHC measures and later dementia assessment. Although self-reported highest level of education was captured at follow up, this variable related to early life and was considered as predictive. Education was analyzed as a categorical variable (i.e., primary school level only, some or all secondary school, or post school) and as a dichotomous variable in modeling (i.e., primary vs. secondary/post school).

Continuous outliers for the earliest and latest WPHC measures, by CIND/Dementia status, were identified using Quartiles 1 and 3 (i.e., Q1 and Q3) and the Interquartile Range (i.e., IQR). Values that were lower than Q1-(IQR^*^1.5) or greater than Q3+(IQR^*^1.5) were considered outliers and winsorized by replacing these values with the next lowest or highest non-outlying value (25, 26). Most continuous variables had five or fewer outliers, with the exception of GGT, UACR, and glucose (see Supplementary Table 2).

### Follow Up/Outcome Variables

The main outcome measure was a diagnosis of “normal,” “CIND” or dementia from the Dementia Prevalence Survey. This variable represented a consensus diagnosis, made by a panel of Geriatricians and an Older Person's Psychiatrist, who blind reviewed results from the comprehensive geriatrician assessments to obtain consensus diagnoses based on criteria from the Diagnostic and Statistical Manual for Mental Disorders, 4th Edition (DSM IV-TR) (18). Participants were classified as normal cognition, dementia, or CIND. The latter group comprised people who met DSM IV-TR criteria of Cognitive Disorder-Not Otherwise Specified (NOS) or Amnestic Disorder-NOS, such as cognitive decline without significance impact of activities of daily living. The full details of this method have been published elsewhere (11). While dementia and CIND are different, both states are characterized by an objective decrease in cognitive functioning relative to age expectations (18), and share similar risk factors (3).

### Statistical Analyses

**Figure 2:**
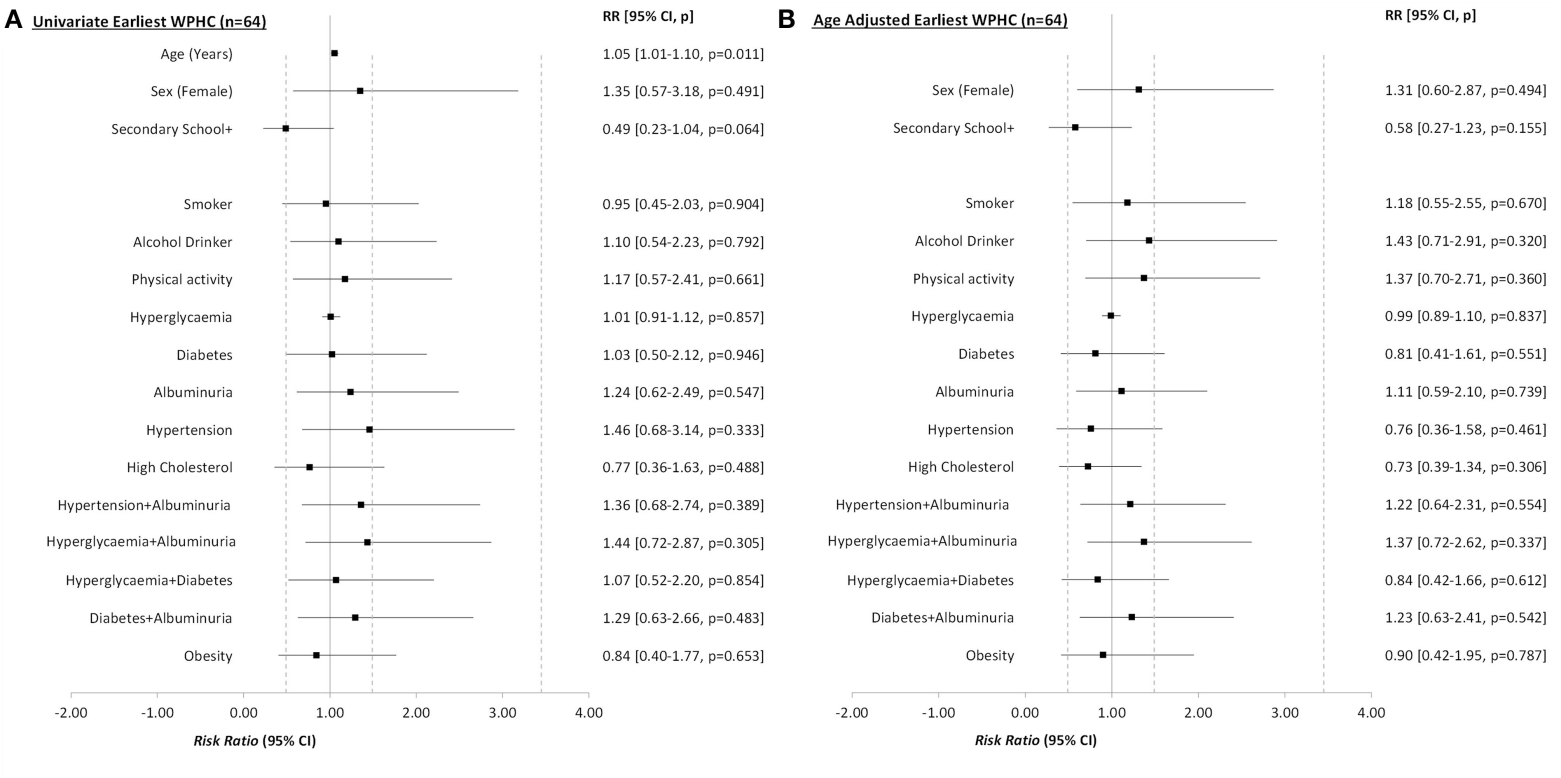
Univariate **(A)** and age adjusted **(B)** risk ratios (RR) from generalized linear model analyses of the earliest risk and protective factors for Cognitive Impairment Not Dementia (CIND) or dementia, among 64 Aboriginal and Torres Strait Islander residents who participated in the Well Person's Health Check (WPHC) (1998/2000, 2005/2006), by dementia status at follow up (2015–2018). Secondary School+, Highest education is secondary school or further.

**Figure 3:**
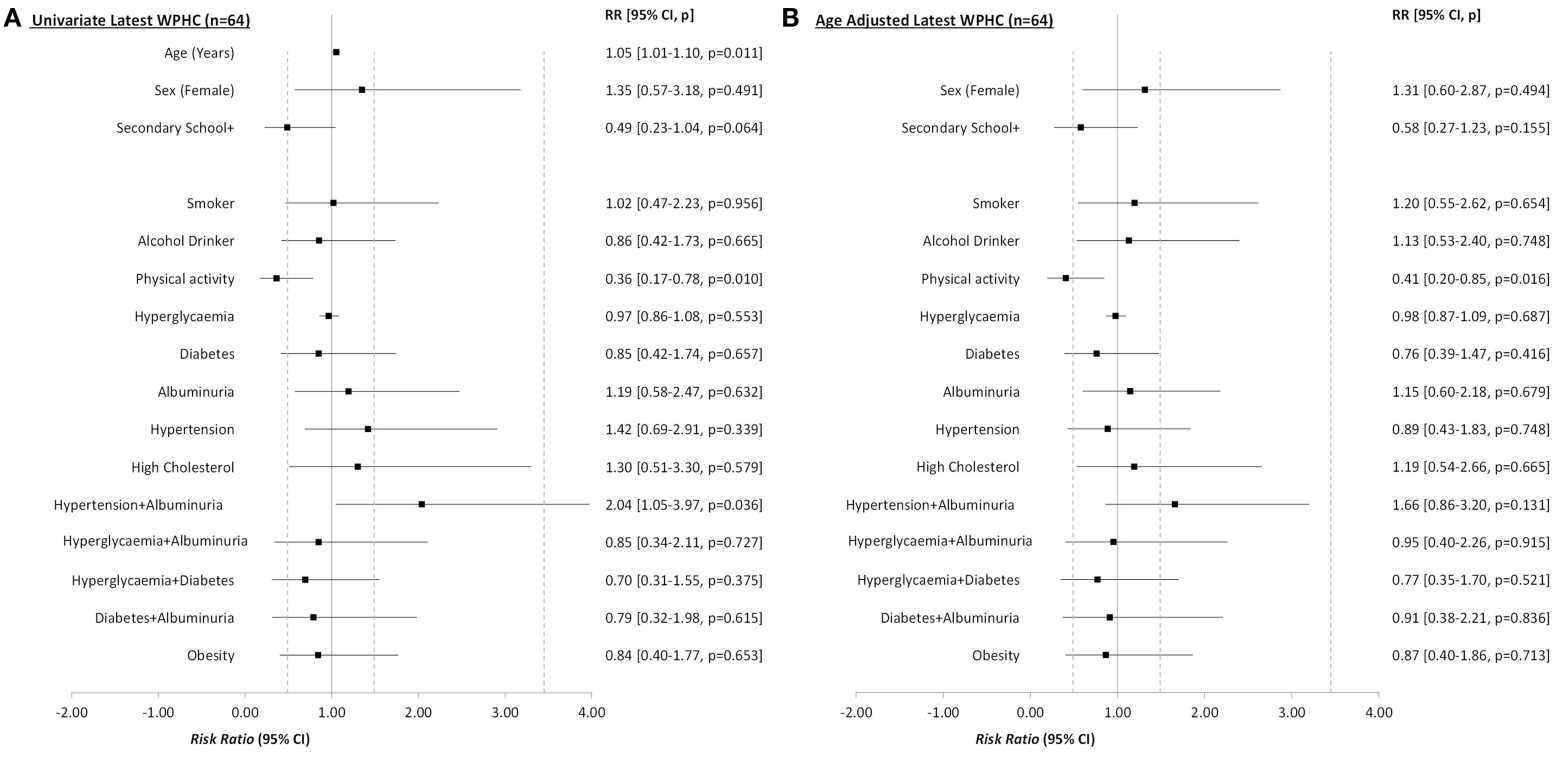
Univariate **(A)** and age adjusted **(B)** risk ratios (RR) from generalized linear model analyses of the latest risk and protective factors for Cognitive Impairment Not Dementia (CIND) or dementia, among 64 Aboriginal and Torres Strait Islander residents who participated in the Well Person's Health Check (WPHC) (1998/2000, 2005/2006), by dementia status at follow up (2015–2018). Secondary School+, Highest education is secondary school or further.

All analyses were undertaken using the Stata 15 software package. Categorical variables were tested against the outcome measure using Pearson chi-square tests for independence or Fisher's Exact tests for expected cell counts <5. Cramer's V was used for effect sizes for categorical analyses (Table 1; Supplementary Table 3). Continuous variables were assessed for normality and examined using mean and Standard Deviation (i.e., SD or ±) when normally distributed or otherwise as median (Interquartile Range—IQR), with appropriate tests of significance (e.g., Independent samples *t*-test or Kruskal-Wallis rank sum tests). Effect sizes were calculated using Cohen's D and Rosenthal's Z score conversion, respectively (Table 1; Supplementary Table 3). Univariate generalized linear modeling was undertaken to create risk ratios (RRs), with CIND/Dementia as a dichotomized outcome and WPHC measures as predictor variables. Age was significantly associated with many WPHC risk factors and was also the most prominent risk factor for CIND/Dementia. To address this confounding, all modeling analyses were also adjusted for age (i.e., aRR) at time of the dementia assessment (Table 2; Figures 2, 3; Supplementary Table 4). *P*-values <0.05 were considered statistically significant.

**Table 1:** Earliest recorded risk and protective factors for Cognitive Impairment Not Dementia (CIND) or dementia, among 64 Aboriginal and Torres Strait Islander residents who participated in the Well Person's Health Check (WPHC) (1998/2000, 2005/2006), by dementia status at follow up (2015–2018).

**Risk and protective variables**	**No—CIND/dementia (*****n*** **=** **43)**			**Yes—CIND/dementia (*****n*** **=** **21)**			**Tests of significance**	
	***N***	**Mean, Med. (%)**	**sd, iqr**	***N***	**Mean, Med. (%)**	**sd, iqr**	***p***	**Effect**
Age (Ax.)^a^	43	63.3	(9.9)	21	71.0	(9.9)	0.005	−0.775
Age WPHC^a^	43	46.6	(10.3)	21	53.9	(10.3)	0.010	−0.712
Sex (Female)^c^	29	(67.4)		16	(76.2)		0.472	0.090
School								
Primary^d^	11	(25.6)		9	(50.0)		0.086	0.283
Secondary	15	(34.9)		2	(11.1)			
Post school	17	(39.5)		7	(38.9)			
Alcohol drinker	21	(48.8)		11	(52.4)		0.790	0.033
Smoker^d^	15	(34.9)		7	(33.3)		0.902	−0.015
Physical activity^d^	14	(32.6)		8	(38.1)		0.781	0.055
BMI≥30^c^	31	(72.1)		14	(66.7)		0.656	−0.056
HBP^c^	23	(53.5)		14	(66.7)		0.316	0.125
High chol.^c^	34	(79.1)		15	(71.4)		0.498	−0.085
Alb.^d^	14	(35.0)		9	(42.9)		0.587	0.077
Diab.^d^	16	(37.2)		8	(38.1)		0.945	0.009
HBG^c^	20	(47.6)		11	(52.4)		0.722	0.045
HBG+Diab.^d^	15	(35.7)		8	(38.1)		0.853	0.023
HBG+Alb.^d^	10	(25.6)		8	(38.1)		0.315	0.130
HBP+Alb.^d^	11	(27.5)		8	(38.1)		0.396	0.109
Diab.+Alb.^d^	10	(25.0)		7	(33.3)		0.490	0.088
Weight (kg)^a^	43	92.9	(17.0)	21	83.9	(14.2)	0.040	0.558
BMI^a^	43	33.4	(6.4)	21	32.4	(6.4)	0.550	0.160
Waist Circ.^a^	43	109.5	(13.7)	21	105.2	(11.4)	0.220	0.330
Vegetables^b^	43	1.0	(0.0–2.0)	21	1.0	(1.0–2.0)	0.223	−0.152
Fruit^b^	43	1.0	(0.0–2.0)	21	1.0	(0.0–2.0)	0.766	−0.037
Systolic BP^a^	43	131.3	(16.4)	21	145.2	(27.2)	0.034	−0.680
Diastolic BP^a^	43	72.1	(12.4)	21	78.3	(13.9)	0.077	−0.478
HbA1c^b^	33	6.6	(6.1–7.8)	10	6.4	(6.2–7.2)	0.634	0.073
Cholesterol^a^	43	5.2	(1.0)	21	5.2	(1.1)	0.760	0.082
Triglycerides^b^	43	1.9	(1.0–2.5)	21	1.6	(0.9–2.2)	0.154	0.178
VLDL^b^	43	0.9	(0.5–1.1)	18	0.7	(0.4–0.8)	0.044	0.258
LDL^a^	43	3.3	(0.8)	18	3.1	(3.1)	0.388	0.244
HDL^a^	43	1.1	(0.2)	21	1.2	(0.2)	0.076	−0.481
GGT^b^	43	26.0	(22.0–35.0)	21	26.0	(18.0–36.0)	0.626	0.061
Glucose^b^	42	5.4	(4.9–7.9)	21	5.7	(4.9–9.2)	0.635	−0.060
UACR^b^	40	0.9	(0.5–4.4)	21	2.4	(0.7–12.8)	0.181	−0.171

*WPHC, Well Person's Health Check, earliest recorded information was from the first WPHC screen if the person attended, or the second screen if the person did not attend the first screen; Age (Ax.), Age at dementia screen; Physical activity, Adequate levels (≥20 min moderate exercise, ≥5 times in the last week); kg, kilograms; BMI, Body Mass Index; BP, Blood Pressure; HBP, Hypertension; High chol., High cholesterol; Alb., Albuminuria (UACR≥2.5 mg/mmol for males and ≥3.5 for females); HBG, Hyperglycemia (fasting glucose ≥ 5.5 mmol/l); Diab., Diabetes; HbA1c, glycated hemoglobin (%); Waist Circ., Waist Circumference (centimeters, cm); Vegetables, Vegetable Quantity; Fruit, Fruit Quantity; HDL, High Density Lipoprotein; LDL, Low-Density Lipoprotein; GGT, Gamma-Glutamyl Transferase; VLDL, Very Low-Density Lipoprotein; Glucose, fasting glucose (mmol/l); UACR, Urinary Albumin Creatinine Ratio*.

a *Continuous—normally distributed, mean (Standard Deviation, sd), t-test for differences, Effect Size = Cohen's D*.

b *Continuous—not normally distributed, median (Interquartile Range, iqr), ranksum test for differences, Effect Size = Rosenthal (1994) Z score conversion (r = Z/√N)*.

c *Categorical—adequate cell size, n (proportion %), Chi^2^ test for differences, Effect Size = Cramer's V*.

d *Categorical—expected cell size <5, n (proportion %), Fisher's Exact test for differences, Effect Size = Cramer's V*.

**Table 2:** Univariate and age adjusted risk ratios (RR) from generalized linear model analyses of earliest risk and protective factors for Cognitive Impairment Not Dementia (CIND) or dementia, among 64 Aboriginal and Torres Strait Islander residents who participated in the Well Person's Health Check (WPHC) (1998/2000, 2005/2006), by dementia status at follow up (2015–2018).

**Risk and protective variables**	**Univariate**			**Age adjusted**		
	**RR**	**95% CI**	***p***	**RR**	**95% CI**	***p***
Age (Ax.)	1.05	(1.01–1.10)	0.011			
Age WPHC	1.05	(1.01–1.09)	0.020			
Sex (Female)	1.35	(0.57–3.18)	0.491	1.31	(0.60–2.87)	0.494
School						
Primary	Reference			Reference		
Secondary/post	0.49	(0.23–1.04)	0.064	0.58	(0.27–1.23)	0.155
Alcohol drinker	1.10	(0.54–2.23)	0.792	1.43	(0.71–2.91)	0.320
Smoker	0.95	(0.45–2.03)	0.904	1.18	(0.55–2.55)	0.670
Physical activity	1.17	(0.57–2.41)	0.661	1.37	(0.70–2.71)	0.360
BMI≥30	0.84	(0.40–1.77)	0.653	0.90	(0.42–1.95)	0.787
HBP	1.46	(0.68–3.14)	0.333	0.76	(0.36–1.58)	0.461
High chol.	0.77	(0.36–1.63)	0.488	0.73	(0.39–1.34)	0.306
Alb.	1.24	(0.62–2.49)	0.547	1.11	(0.59–2.10)	0.739
Diab.	1.03	(0.50–2.12)	0.946	0.81	(0.41–1.61)	0.551
HBG	1.01	(0.91–1.12)	0.857	0.99	(0.89–1.10)	0.837
HBG+Diab.	1.07	(0.52–2.20)	0.854	0.84	(0.42–1.66)	0.612
HBG+Alb.	1.44	(0.72–2.87)	0.305	1.37	(0.72–2.62)	0.337
HBP+Alb.	1.36	(0.68–2.74)	0.389	1.22	(0.64–2.31)	0.554
Diab.+Alb.	1.29	(0.63–2.66)	0.483	1.23	(0.63–2.41)	0.542
Weight (kg)	0.98	(0.96–1.00)	0.016	0.98	(0.95–1.01)	0.160
BMI	0.98	(0.93–1.04)	0.540	0.99	(0.93–1.07)	0.871
Waist Circ.	0.98	(0.96–1.01)	0.165	0.98	(0.95–1.00)	0.075
Vegetables	1.08	(0.86–1.35)	0.505	1.03	(0.83–1.30)	0.766
Fruit	1.00	(0.84–1.19)	0.994	0.97	(0.82–1.16)	0.768
Systolic BP	1.02	(1.01–1.03)	0.007	1.01	(0.99–1.03)	0.320
Diastolic BP	1.02	(1.00–1.05)	0.060	1.01	(0.98–1.04)	0.365
HbA1c	0.75	(0.51–1.08)	0.122	0.79	(0.49–1.28)	0.337
Cholesterol	0.95	(0.66–1.36)	0.766	0.88	(0.62–1.26)	0.490
Triglycerides	0.68	(0.46–0.98)	0.040	0.63	(0.41–0.97)	0.034
VLDL	0.26	(0.09–0.73)	0.010	0.22	(0.07–0.69)	0.009
LDL	0.79	(0.47–1.31)	0.354	0.75	(0.46–1.25)	0.274
HDL	4.83	(0.92–25.29)	0.062	3.24	(0.53–20.01)	0.205
GGT	1.00	(0.97–1.03)	0.828	1.00	(0.97–1.03)	0.954
Glucose	1.07	(0.96–1.19)	0.251	1.04	(0.92–1.17)	0.547
UACR	1.08	(1.04–1.11)	0.000	1.05	(1.01–1.09)	0.006

*WPHC, Well Person's Health Check, earliest recorded information was from the first WPHC screen if the person attended, or the second screen if the person did not attend the first screen. RR, Relative Risk; Age (Ax.), Age at dementia screen; Physical activity, Adequate levels (≥20 min moderate exercise, ≥5 times in the last week); kg, kilograms; BMI, Body Mass Index; BP, Blood Pressure; HBP, Hypertension; High chol., High cholesterol (###); Alb., Albuminuria (###); HBG, Hyperglycemia (fasting glucose ≥ 5.5 mmol/l); Diab., Diabetes; HbA1c, glycated hemoglobin (%); Waist Circ., Waist Circumference (centimeters, cm); Vegetables, Vegetable Quantity; Fruit, Fruit Quantity; HDL, High Density Lipoprotein; LDL, Low-Density Lipoprotein; GGT, Gamma-Glutamyl Transferase; VLDL, Very Low-Density Lipoprotein; Glucose, fasting glucose (mmol/l); UACR, Urinary Albumin Creatinine Ratio*.

Multivariable modeling was limited by the small sample size. To accommodate this limitation, bootstrapping with the Stata command “swboot” was used to inform variable selection (Table 3). First, variables that were at or below *p* = 0.200 in univariate generalized linear modeling were selected into a single multivariate model (Model 1). Non-significant variables or those highly correlated with other variables (i.e., Pearson's r ≥ 0.3) were removed, and the remaining variables entered into the “swboot” command with 200 and 100 repetitions for earliest and latest risk factors, respectively. Variables that were present in 25% of the bootstrap models were entered into Model 2, and the bootstrap method was repeated. Non-significant variables, that were highly correlated and had low presentations in the bootstrapping (i.e., <50%) were then removed, and the remaining variables were entered into the final model (Model 3) (Table 3). Multicollinearity was assessed with the STATA command “coldiag2” and presented as a Condition Index, with values 10–30 indicate moderate collinearity (27). Although age lost significance during modeling, this was due to collinearity with other variables and once these variables were removed, age remained significant. Analyses were repeated with subsets of the data limited to participants aged <60 years at the time of baseline assessment, to examine the potential effect of older participants at baseline.

**Table 3:** Multivariate risk ratios from generalized linear modeling, with bootstrapping variable selection, for earliest and latest recorded risk and protective factors for Cognitive Impairment Not Dementia (CIND) or dementia, among 64 Aboriginal and Torres Strait Islander residents who participated in the Well Person's Health Check (WPHC) (1998/2000, 2005/2006).

**Risk and protective factors**	**Model 1 (*****n*** **=** **40)**			**B/Strap**	**Model 2 (*****n*** **=** **58)**			**B/Strap**	**Model 3 (*****n*** **=** **61)**		
	**RR**	**95% CI**	***p***		**RR**	**95% CI**	***p***		**RR**	**95% CI**	***p***
**Earliest WPHC measures**				**(#/200)**				**(#/200)**			
Age (Ax.)	1.36	(1.09–1.70)	0.006	107	1.03	(0.99–1.08)	0.140	109	1.06	(1.02–1.10)	0.005
Primary school	Ref										
Secondary/post school	0.09	(0.01–0.85)	0.035	98	0.43	(0.22–0.82)	0.011	114	0.38	(0.19–0.76)	0.006
Waist Circ. (cm)	0.78	(0.66–0.94)	0.007	174	0.94	(0.91–0.97)	0.000	175	0.95	(0.93–0.98)	0.000
Triglycerides	555.56	(9.00–34,284.00)	0.003	171	0.39	(0.25–0.61)	0.000	164	0.43	(0.28–0.66)	0.000
Systolic BP	1.07	(1.02–1.13)	0.010	84	1.02	(1.00–1.04)	0.121	80			
UACR	1.19	(1.04–1.36)	0.011	69	1.04	(1.00–1.08)	0.051	49			
HDL	0.33	(0.01–14.94)	0.568	36							
Diastolic BP	0.86	(0.79–0.94)	0.001								
VLDL	0.00	(0.00–0.00)	0.002								
Weight (kg)	1.09	(0.99–1.19)	0.070								
HbA1c	1.65	(0.71–3.83)	0.243								
**Model information**											
Collinearity Index	78.9				34.12				28.02		
BIC	−95.24				−181.02				−199.4		
**Latest WPHC measures**	**Model 1 (** ***n** **=** **55*** **)**			**(#/100)**	**Model 2 (*****n*** **=** **61)**			**(#/200)**	**Model 3 (*****n*** **=** **61)**		
Age (Ax.)	1.05	(1.01–1.09)	0.015	51	1.04	(1.01–1.07)	0.009	86	1.04	(1.01–1.07)	0.006
Primary school	Ref										
Secondary/post school	0.16	(0.04–0.67)	0.012	75	0.36	(0.17–0.75)	0.007	147	0.37	(0.17–0.76)	0.007
Physical activity	0.20	(0.09–0.44)	0.000	92	0.27	(0.14–0.54)	0.000	196	0.26	(0.13–0.52)	0.000
Weight (kg)	0.92	(0.85–0.99)	0.026	94	0.94	(0.92–0.97)	0.000	193	0.94	(0.91–0.97)	0.000
Triglycerides	1.20	(0.00–472.47)	0.953	68	0.51	(0.29–0.89)	0.017	115	0.50	(0.29–0.86)	0.012
Vegetables	1.02	(0.83–1.25)	0.873	26	0.97	(0.80–1.18)	0.772	64			
Glucose	1.01	(0.64–1.59)	0.980	14							
GGT	0.95	(0.88–1.02)	0.173								
Waist Circ.	0.96	(0.88–1.05)	0.392								
BMI	1.16	(0.84–1.60)	0.368								
HBP and Alb.	0.97	(0.38–2.48)	0.950								
VLDL	0.08	(0.00–15,904.78)	0.683								
**Model information**											
Collinearity index	112.27				31.23				29.46		
BIC	−151.60				−197.40				−201.5		

*WPHC, Well Person's Health Check. Earliest recorded information was from the first WPHC screen if the person attended, or the second screen if the person did not attend the first screen. Latest recorded information was from the second WPHC screen if the person attended, or the first screen if the person did not attend the second screen; RR, Relative Risk; Age (Ax.), Age at dementia screen; kg, kilograms; BMI, Body Mass Index; HbA1c, glycated hemoglobin (%); Waist Circ., Waist Circumference (centimeters, cm); VLDL, Very Low-Density Lipoprotein; BP, Blood Pressure; HDL, High Density Lipoprotein; UACR, Urinary Albumin Creatinine Ratio; GGT, Gamma-Glutamyl Transferase; Vegetables, Vegetable Quantity; HBP, Hypertension; Glucose, fasting glucose (mmol/l); Alb., Albuminuria (UACR ≥ 2.5 mg/mmol for males and ≥3.5 for females); Physical activity, Adequate levels (≥20 min moderate exercise, ≥5 times in the last week); BIC, Bayesian information criterion*.

## Results

### Data Linkage

Figure 1 shows that 65 participants in WPHC and Dementia Prevalence Study were linked over four rounds of probabilistic linkage based on multiple combinations of unique identifiers. The weights assigned to each unique identifier and the thresholds used to determine a successful linkage are described in Supplementary Figure 1. One participant was missing dementia diagnosis status at follow up and was excluded from further analyses. The final linked dataset comprised 64 individuals, of which 9.6% (*n* = 6) were diagnosed with dementia between 2015 and 2018, 23.4% (*n* = 15) with CIND, and 67.2% (*n* = 43) were cognitively normal. Due to the small sample size, participants with dementia or CIND were grouped together for the main analyses (*n* = 21, 32.8%). The participants were living in eight communities in the Torres Strait and five in the Northern Peninsula Area. While most were from geographical locations with larger populations, their overall distribution was not representative of census population estimates for the entire region (data not tabled).

Figure 1 shows the majority (59%) of linked participants took part in both WPHC assessments, although this was lower for participants who later had CIND/Dementia (42.9%) compared with those without (67.4%) (*p* = 0.019). Figure 1 also provides the average time in years to follow up dementia assessment by participation in a WPHC screen.

### Description of Risk Factors–Earliest WPHC Assessment

The mean age of the sample was 48.9 years (SD = 10.8, range = 25–68) at baseline and was mostly female (70.3%). Table 1 shows the distribution of risk factors at the earliest WPHC assessment, by later CIND/Dementia status. Participants with CIND/Dementia were ~10 years older at the earliest WPHC assessment (mean = 53.9 ± 10.3) and dementia screen (71.0 ± 9.9) compared with cognitively normal participants (46.6, *p* = 0.010 and 63.3, *p* = 0.005, respectively). Participants with CIND/Dementia were more likely to only have a primary (50.0%) school education compared with those who were cognitively normal, although significance was only at trend level (25.6%, *p* = 0.086). There were high rates of hypertension, diabetes, albuminuria, hyperglycemia, and combinations of these risk factors in both groups. While these factors were generally more frequent among those who later developed CIND/Dementia, the effect sizes for differences were modest. Mean body weight (83.9 kg, range = 60.3–106.7) and median VLDL (0.7, range = 0.3–1.1) were significantly lower in the CIND/Dementia group compared with the cognitively normal group (92.9 kg, range = 66.7–128.2, *p* = 0.040 and 0.9, range = 0.3–1.8, *p* = 0.044, respectively). Mean systolic blood pressure (145.2 ± 27.2) in the CIND/Dementia group was significantly higher compared with the cognitively normal group (mean = 131.3 ± 16.4, *p* = 0.034).

### Risk Ratios—Univariate and Adjusted for Age

Generalized linear modeling (Table 2; Figures 2A,B) showed age at the WPHC was positively associated with dementia risk (aRR 1.05, 95% CI 1.01–1.10, *p* = 0.011). While secondary or post-school education appeared to be protective against later life CIND/Dementia compared with primary school only, this effect was attenuated after adjusting for age (aRR = 0.58, 95% CI 0.27–1.23, *p* = 0.155). After adjusting for age, waist circumference and triglycerides were lower among people who later developed CIND/Dementia (aRR = 0.98, 95% CI 0.95–1.00, *p* = 0.075 and aRR = 0.63, 95% CI 0.41–0.97, *p* = 0.034, respectively), while UACR was higher (aRR 1.05, 95% CI 1.01–1.09, *p* = 0.006).

### Modeling Risk Factors—Earliest WPHC Assessment

Variables significant at the *p* ≤ 0.200 level during univariate analyses were age and highest education dichotomized, and the earliest measures for body weight, waist circumference, systolic and diastolic blood pressure, HDLC, VLDL, triglycerides, HbA1c, and UACR (see Table 2). These were entered into a single multivariate analysis (i.e., Model 1) for bootstrapping assisted variable selection (Table 3). The variables diastolic blood pressure, HbA1c, VLDL, and weight were removed from further modeling due to low significance and/or collinearity with other variables. Bootstrapping removed HDLC (i.e., variable selection <50). The remaining variables were analyzed in a multivariate model (Model 2) and systolic blood pressure and UACR were then removed due to non-significance, low selection in bootstrapping, and high correlation with age. In the final model (Model 3), which had moderate multicollinearity (i.e., Collinearity Index = 28.02) increasing age was significantly associated with CIND/Dementia risk, more education was protective, and waist circumference and triglycerides were again significantly lower among people who later developed CIND/Dementia. These variables were also selected frequently in bootstrapping (i.e., >50% of models).

### Risk Factors—Latest WPHC Assessment

At the time of the latest WPHC measures, the mean age of participants was 52.9 (SD = 11.4, range = 25–75). Adequate levels of physical activity were protective for later CIND/Dementia risk (aRR 0.41, 95% CI 0.20–0.85, *p* = 0.016) (Supplementary Tables 3, 4; Figure 3). Bodyweight and VLDL were negatively associated with CIND/Dementia (*p* = 0.042 and *p* = 0.019 respectively). After adjusting for age, albuminuria was the only vascular factor with positive risk ratios at both the latest (Figure 3B) and earliest (Figure 2B) WPHC assessments. This trend also held when albuminuria was comorbid with hypertension. However, these ratios were modest and non-significant, reflecting the small cell sizes and wide confidence intervals. Multivariate modeling of latest WPHC measures (Table 3), with bootstrapping and removal of variables for collinearity, produced a final model where age was significantly positively associated with CIND/Dementia risk, while more education, body weight, and triglycerides were significantly negatively associated with CIND/Dementia risk. In this model, sufficient physical activity measured at the latest WPHC was protective for later CIND/Dementia. However, this was inconsistent with the trend from the earliest WPHC data (see Figures 2B, 3B).

### *Post-hoc* Analyses

The earliest measures of waist circumference and body weight were examined in *post-hoc* analyses. Waist circumference was not significantly associated with other non-anthropometric baseline study variables. Weight was negatively correlated with age (Pearson's R = −0.285, *p* = 0.023). For comparison, all the main analyses with the earliest data were repeated on 51 participants aged <60 years at the baseline measures. The main findings for age, education, weight, and triglycerides were replicated (Supplementary Table 5).

## Discussion

This study linked health check data for 64 Torres Strait Islander and Aboriginal peoples living in remote communities with their results on a single dementia assessment 10–20 years later. A third (*n* = 21) of the participants had either CIND or dementia (*n* = 15 and *n* = 6, respectively) at follow up. Older age was the most prominent risk factor. More education and adequate levels of self-reported physical activity at one timepoint measure had some evidence of being protective, as they were frequently selected in bootstrapping and statistically significant the final models, albeit with moderate levels of multicollinearity. After adjusting for age, albuminuria, with or without comorbid hypertension, was the only vascular factor associated with later risk of CIND/Dementia from both the earliest and latest health check information available. However, these risk ratios were modest, and it did not reach statistical significance during any analyses. Unexpectedly, waist circumference, weight, triglycerides, and VLDL were significantly lower among people who later developed CIND/Dementia. All results should be interpreted in the context of the small sample size. This limitation made for wide confidence intervals and inconsistent associations between the earliest and latest health check measures and later CIND/dementia status.

In this small, linked cohort, more education and adequate levels of physical activity were the two most suggestive protective factors for CIND/dementia. These were defined as education beyond primary school and moderate physical activity for ≥20 min for ≥5 days in the previous 7 days, respectively. The protective effect of these factors is well-established in the broader literature on dementia prevention (3, 28, 29). For Aboriginal peoples in Australia, more school education (12), occupational complexity, and lifelong learning (30) have also been identified as protective for dementia. To our knowledge, no similar previous research has examined the long-term protective effect of exercise on dementia risk among Aboriginal and Torres Strait Islander Australians. The current study contributes new knowledge by suggesting education and physical activity remain protective for this population after statistically adjusting for competing health risks. As these are modifiable risk factors, ongoing investment in education and improving physical activity levels across the lifespan may represent opportunities to reduce the future burden of dementia as this population ages.

After adjusting for age, albuminuria, with or without comorbid hypertension, was the most suggestive vascular risk measures for later CIND/Dementia in the current study. Although small numbers made for wide confidence intervals and non-significant associations. Albuminuria is proposed to be a sensitive biomarker of systemic microcirculatory dysfunction, including damage to the microvasculature of the brain (23). Although our results require support with more data, they concur with an association between chronic kidney disease and dementia among residents of the Torres Strait and Northern Peninsula Area identified elsewhere (17). In terms of international applicability, in populations where both dementia and kidney disease are prevalent, the impact of kidney health in earlier life may be important. To our knowledge, no previous studies have included measures of albuminuria as a risk for dementia in First Nations populations. This has occurred despite multiple systematic reviews linking it with CIND/Dementia risk in other populations internationally (23) and with cardiovascular disease risk in Aboriginal and Torres Strait Islander peoples in Australia (20).

The other baseline vascular risks, such as diabetes, had no clear relationship with later CIND/Dementia. This finding is consistent with cross-sectional research of dementia among Aboriginal Australians (8). The null relationship is thought to reflect the high prevalence of these risk factors within this population, the correspondingly low sensitivity of these measures to differentiate dementia risk, and lack of information about intervening treatment (e.g., medication and adherence).

Several results of this study were unexpected. Participants who had CIND/Dementia at follow up had lower levels of triglycerides at baseline. Triglycerides are lipids that store and transport energy in the body. High levels of triglycerides are generally associated with increased risk of cognitive impairment over time (31) and non-Alzheimer's dementia (32), although evidence is sparse and non-associations have also been reported (31, 33). A few cross-sectional studies have reported similar results to ours, where low levels of triglycerides were associated with dementia (34, 35). A 2020 study showed low levels of a certain category of triglycerides, polyunsaturated fatty acid-containing triglycerides (PUTGs), were associated with cognitive impairment, Alzheimer's Disease, and increased neural atrophy (36). The authors proposed that reduced serum PUTGs may also result in decreased availability of neuroprotective polyunsaturated fatty acids. Our study contributes some longitudinal information to this discussion and highlights the importance of ongoing examination of triglycerides as a novel biomarker for dementia risk.

Waist circumference and body weight at baseline were negatively associated with risk of later CIND/Dementia. These results contrast most longitudinal research, where higher anthropometric measures in midlife are risks for developing dementia (3). Our *post-hoc* analyses did not find associations between these anthropometric measures and other study variables, which would explain our counterintuitive findings. Similar research among older Aboriginal Australians has found low BMI (i.e., ≤ 25) was associated with dementia (30), and lower BMI measured at follow-up was associated with a decline from cognitively normal to CIND/Dementia over 5 years (13). The associations found in these studies were thought to reflect comorbid frailty in dementia. Our results may reflect an earlier stage in this process; however, this should be confirmed with larger longitudinal studies in similar populations.

### Limitations and Strengths

The small sample size was below the number required based on sample size calculations. This reduced the generalizability of our findings, which should be interpreted with caution. As there were a small number of participants with CIND or dementia, we were required to combine these two distinct syndromes into a single dichotomous outcome measure, which also reduced generalizability. The small sample size also limited modeling reliability, resulted in moderate collinearity (i.e., Condition Index 10–30) and likely contributed to inconsistent associations between the earliest and latest health check measures and later CIND/dementia status. Further, the baseline data did not include assessments of cognition and included some older participants, who may have had prodromal dementia 10–20 years before follow-up assessment. However, our comparative analyses limited to participants aged <60 years at baseline produced the same main findings, suggesting that CIND/dementia at baseline was unlikely. The population is also culturally unique, which should be considered when generalizing findings to other First Nations populations, including in Australia. The baseline WHPC measures were also limited to “snapshots” of an individual's health, and no long-term information about medication prescribing, treatment, or change over time was available. Despite these limitations, this modest study is the first to describe long-term risk factors in a population with a high prevalence of CIND/Dementia and is the first to leverage data linkage of disparate studies to reduce participant burden. While the baseline measures were not comprehensive, they are the same measures that are collected routinely in primary care in Australia, so have real world applicability. The results may also have some global relevance to other populations that also experience high lifetime exposure to potentially modifiable dementia risks.

## Conclusion

In a Torres Strait Islander and Aboriginal population in North Queensland, Australia, where CIND/dementia is highly prevalent in older age, our preliminary findings suggest that early life education and adequate levels of physical activity in adult life may be protective against CIND/dementia over time, after statistical adjustment for other factors measured earlier in life. Among the vascular risk measures available, albuminuria, with or without comorbid hypertension, had the most consistent risk ratio for later CIND/dementia. However, these ratios were modest, with small cell sizes and non-significant associations. These results suggest middle-aged community members with low levels of formal education and physical activity, with albuminuria and comorbid hypertension, may be at heightened risk of developing cognitive impairment within the next 10–20 years. These individuals may benefit from targeted clinical and lifestyle interventions to improve the chance of preventing or delaying CIND/dementia.

## Data Availability Statement

The data that support the findings of this study are not publicly available due to privacy or ethical restrictions. The data are available on request from the corresponding author. Additional institutional approvals, such as ethics approval, would be required to enable sharing of these data. Requests to access the datasets should be directed to fintan.thompson@jcu.edu.au.

## Ethics Statement

This study was reviewed and approved by the Far North Queensland Human Research Ethics Committee (HREC/18/QCH92-1262). The Ethics Committee waived the requirement of written informed consent for participants to have their data linked.

## Author Contributions

FT linked the data, analyzed the linked dataset, and drafted the manuscript. SR and LH advised on the interpretation of the results and drafting of the manuscript. AE provided statistical advice. ST provided cultural advice and assisted with drafting the manuscript. RQ provided advice on the context of the research. ES and RM provided oversight of the project and clinical content of the manuscript. All authors reviewed and contributed to the content of the final manuscript.

## Funding

FT was supported with a Postgraduate Scholarship from the Australian National Health and Medical Research Council (NHMRC) (GNT1191144).

## Conflict of Interest

The authors declare that the research was conducted in the absence of any commercial or financial relationships that could be construed as a potential conflict of interest.

## Publisher's Note

All claims expressed in this article are solely those of the authors and do not necessarily represent those of their affiliated organizations, or those of the publisher, the editors and the reviewers. Any product that may be evaluated in this article, or claim that may be made by its manufacturer, is not guaranteed or endorsed by the publisher.
