# Supplementary material for: Using Health Check Data to Understand Risks for Dementia and Cognitive Impairment Among Torres Strait Islander and Aboriginal Peoples in Northern Queensland—A Data Linkage Study
Source: Front Public Health. 2022 Feb 16;10:782373. doi: 10.3389/fpubh.2022.782373 (PMC8888447; doi:10.3389/fpubh.2022.782373)
Supplement: Supplementary file 1 [file Data_Sheet_1.docx]

**Supplementary Figure 1 - Description of probabilistic linkage of datasets from the Well Person’s Health Check (WPHC) (1998/2000, 2005/2006) and the Dementia Prevalence Survey (2015-2018), by Stages of linkage, with variable weightings**

**
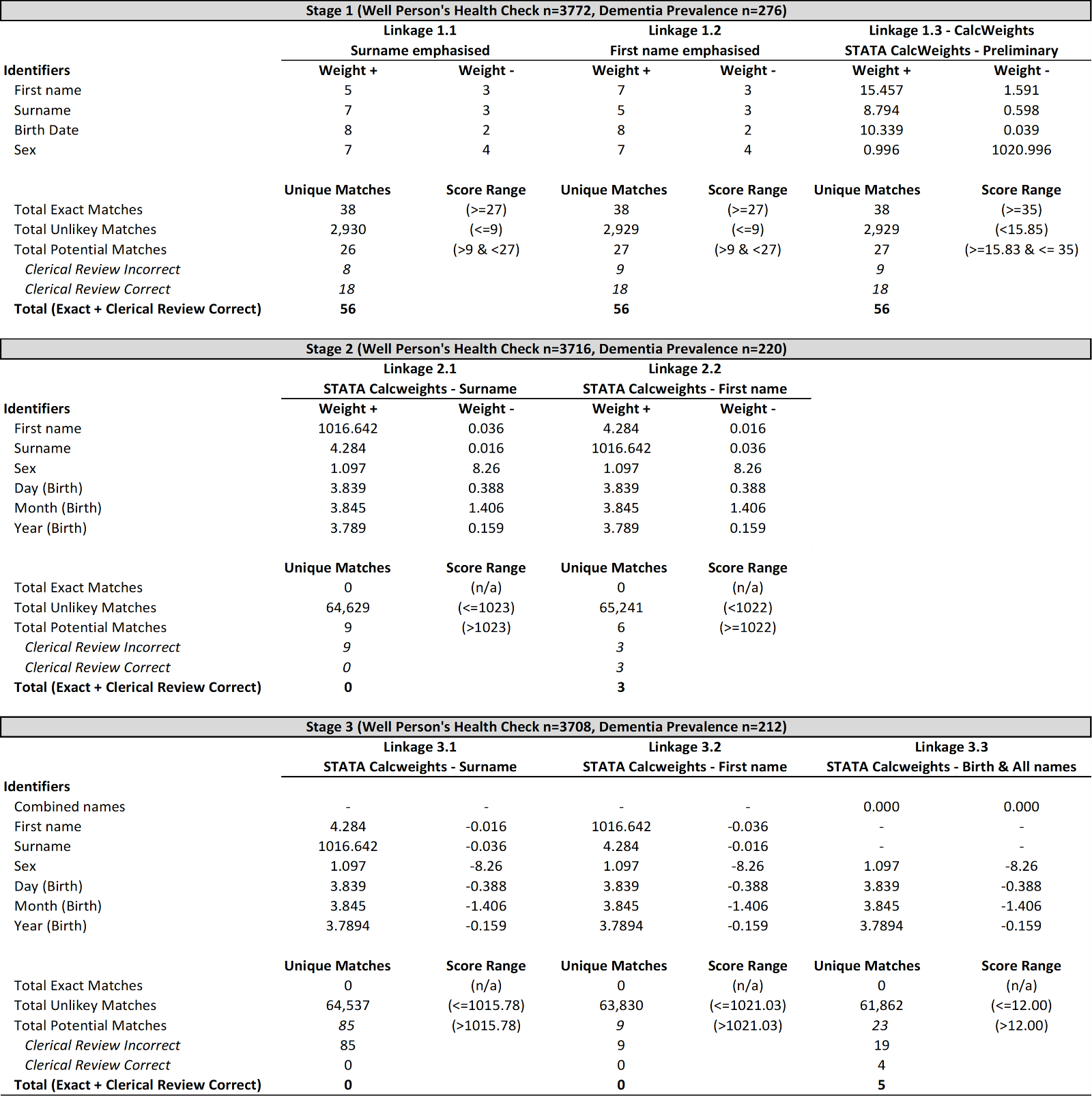
**

**Supplementary Table 1 – Proportion of missing data (%) for study variables, by timing of data collection, among 64 Aboriginal and Torres Strait Islander residents who participated in the Well Person’s Health Check (WPHC) (1998/2000, 2005/2006), by dementia status at follow up (2015-2018)**

| **Variable** | **WPHC Screen** | | **Measures Available** | |
| --- | --- | --- | --- | --- |
|  | **First (1998/2000)** | **Second (2005/2006)** | **Earliest** | **Latest** |
|  | **n=57, (% complete)** | **n=45, (% complete)** | **n=64, (% complete)** | **n=64, (% complete)** |
| **Follow Up - Dementia Assessment (2015-2018)** |  |  |  |  |
| Age |  |  |  | (100.0) |
| Sex |  |  |  | (100.0) |
| Education |  |  |  | (95.3) |
| Diagnosis |  |  |  | (100.0) |
|  |  |  |  |  |
| **Historic Measure - Well Person's Health Check** |  |  |  |  |
| **Health Assessment** |  |  |  |  |
| Age | (100.0) | (100.0) | (100.0) | (100.0) |
| Waist Circumference | (100.0) | (97.8) | (100.0) | (100.0) |
| Weight (kg) | (100.0) | (100.0) | (100.0) | (100.0) |
| Body Mass Index (BMI) | (100.0) | (100.0) | (100.0) | (100.0) |
| Obese (BMI>=30) | (100.0) | (100.0) | (100.0) | (100.0) |
| Smoker | (100.0) | (100.0) | (100.0) | (100.0) |
| Alcohol Drinker | (100.0) | (100.0) | (100.0) | (100.0) |
| Vegetable Quantity | (100.0) | (100.0) | (100.0) | (100.0) |
| Fruit Quantity | (100.0) | (100.0) | (100.0) | (100.0) |
| Physical Activity Sufficient | (100.0) | (100.0) | (100.0) | (100.0) |
| Systolic Blood Pressure | (100.0) | (100.0) | (100.0) | (100.0) |
| Diastolic Blood Pressure | (100.0) | (100.0) | (100.0) | (100.0) |
| **Pathology (continuous)** |  |  |  |  |
| HbA1c | (0.0) | (95.6) | (67.2) | (67.2) |
| Cholesterol | (100.0) | (95.6) | (100.0) | (100.0) |
| Trigylicerides | (100.0) | (95.6) | (100.0) | (100.0) |
| Low Density Lipoprotein (LDL) | (91.2) | (82.2) | (95.3) | (95.3) |
| Very Low Density Lipoprotein (VLDL) | (91.2) | (82.2) | (95.3) | (95.3) |
| High Density Lipoprotein (LDL) | (100.0) | (95.6) | (100.0) | (100.0) |
| Gamma-glutamyl transferase (GGT) | (100.0) | (95.6) | (100.0) | (100.0) |
| Glucose | (100.0) | (93.3) | (98.4) | (98.4) |
| Albumin Creatinine Ratio | (87.7) | (88.9) | (95.3) | (95.3) |
| **Pathology (categorical)** |  |  |  |  |
| High Cholesterol | (100.0) | (100.0) | (100.0) | (100.0) |
| Hypertension | (100.0) | (100.0) | (100.0) | (100.0) |
| Diabetes | (100.0) | (100.0) | (100.0) | (100.0) |
| Albuminuria | (87.7) | (88.9) | (95.3) | (95.3) |
| Hyperglycaemia | (100.0) | (93.3) | (98.4) | (98.4) |
| Hyperglycaemia + Diabetes | (100.0) | (93.3) | (98.4) | (98.4) |
| Hyperglycaemia + Albuminuria | (87.7) | (84.4) | (93.8) | (93.8) |
| Hypertension + Albuminuria | (87.7) | (88.9) | (95.3) | (95.3) |
| Diabetes + Albuminuria | (87.7) | (88.9) | (95.3) | (95.3) |

*Notes: WPHC = Well Person’s Health Check*

**Supplementary Table 2 – Description of suspected outlying values among continuous study measures, by timing of data collection, among 64 Aboriginal and Torres Strait Islander residents who participated in the Well Person’s Health Check (WPHC) (1998/2000, 2005/2006), by dementia status at follow up (2015-2018)**

| **Variable** | **No CIND/Dementia** | | | | | | | |  | **CIND/Dementia** | | | | | | | |
| --- | --- | --- | --- | --- | --- | --- | --- | --- | --- | --- | --- | --- | --- | --- | --- | --- | --- |
|  | **n** | **Cut-off (%)** | | **IQR** | **Boundaries** | | **Outliers (n)** | |  | **n** | **Cut-off (%)** | | **IQR** | **Boundaries** | | **Outliers (n)** | |
|  |  | **25%** | **75%** |  | **Low** | **High** | **Low** | **High** |  |  | **25%** | **75%** |  | **Low** | **High** | **Low** | **High** |
| **Earliest WPHC Measure** |  |  |  |  |  |  |  |  |  |  |  |  |  |  |  |  |  |
| Weight (kg) | 43 | 79.0 | 101.2 | 22.2 | 45.7 | 134.5 | 0 | 2 |  | 21 | 76.9 | 89.9 | 13.0 | 57.4 | 109.4 | 1 | 0 |
| BMI | 43 | 27.8 | 36.6 | 8.8 | 14.5 | 49.9 | 0 | 1 |  | 21 | 29.2 | 36.9 | 7.7 | 17.7 | 48.5 | 0 | 0 |
| Waist Circumference (cm) | 43 | 100.0 | 118.5 | 18.5 | 72.3 | 146.3 | 0 | 1 |  | 21 | 100.0 | 114.5 | 14.5 | 78.3 | 136.3 | 1 | 0 |
| Systolic Blood Pressure | 43 | 118.0 | 145.0 | 27.0 | 77.5 | 185.5 | 0 | 0 |  | 21 | 127.0 | 164.0 | 37.0 | 71.5 | 219.5 | 0 | 0 |
| Diastolic Blood Pressure | 43 | 64.0 | 80.0 | 16.0 | 40.0 | 104.0 | 0 | 0 |  | 21 | 69.0 | 90.0 | 21.0 | 37.5 | 121.5 | 0 | 0 |
| HbA1c | 33 | 6.1 | 7.8 | 1.7 | 3.5 | 10.4 | 0 | 4 |  | 10 | 6.2 | 7.2 | 1.0 | 4.7 | 8.7 | 0 | 1 |
| Cholesterol | 43 | 4.4 | 5.7 | 1.3 | 2.5 | 7.6 | 0 | 1 |  | 21 | 4.5 | 6.0 | 1.5 | 2.3 | 8.3 | 0 | 0 |
| Triglycerides | 43 | 1.0 | 2.5 | 1.5 | -1.3 | 4.8 | 0 | 1 |  | 21 | 0.9 | 2.2 | 1.3 | -1.1 | 4.2 | 0 | 3 |
| VLDL | 43 | 0.5 | 1.1 | 0.6 | -0.4 | 2.0 | 0 | 0 |  | 18 | 0.4 | 0.8 | 0.4 | -0.2 | 1.4 | 0 | 0 |
| LDL | 43 | 2.8 | 3.8 | 1.0 | 1.3 | 5.3 | 0 | 2 |  | 18 | 2.7 | 3.5 | 0.8 | 1.5 | 4.7 | 0 | 0 |
| LDL | 43 | 0.9 | 1.3 | 0.4 | 0.3 | 1.9 | 0 | 0 |  | 21 | 1.0 | 1.3 | 0.3 | 0.6 | 1.7 | 0 | 0 |
| GGT | 43 | 22.0 | 35.0 | 13.0 | 2.5 | 54.5 | 0 | 6 |  | 21 | 18.0 | 36.0 | 18.0 | -9.0 | 63.0 | 0 | 1 |
| Glucose | 42 | 4.9 | 7.9 | 3.0 | 0.4 | 12.4 | 0 | 7 |  | 21 | 4.9 | 9.2 | 4.3 | -1.5 | 15.6 | 0 | 0 |
| Albumin Creatinine Ratio | 40 | 0.5 | 4.4 | 3.9 | -5.4 | 10.3 | 0 | 7 |  | 21 | 0.7 | 12.8 | 12.1 | -17.5 | 31.0 | 0 | 3 |
| **Latest WPHC Measure** |  |  |  |  |  |  |  |  |  |  |  |  |  |  |  |  |  |
| Weight (kg) | 43 | 82.0 | 103.0 | 21.0 | 50.5 | 134.5 | 0 | 2 |  | 21 | 76.0 | 88.4 | 12.4 | 57.4 | 107.0 | 0 | 0 |
| BMI | 43 | 29.1 | 36.6 | 7.5 | 17.8 | 47.9 | 0 | 2 |  | 21 | 28.4 | 34.3 | 5.9 | 19.5 | 43.2 | 0 | 1 |
| Waist Circumference (cm) | 43 | 102.0 | 121.1 | 19.1 | 73.4 | 149.8 | 0 | 1 |  | 21 | 102.0 | 113.0 | 11.0 | 85.5 | 129.5 | 0 | 0 |
| Systolic Blood Pressure | 43 | 120.0 | 137.0 | 17.0 | 94.5 | 162.5 | 0 | 0 |  | 21 | 122.0 | 145.0 | 23.0 | 87.5 | 179.5 | 0 | 1 |
| Diastolic Blood Pressure | 43 | 67.0 | 82.0 | 15.0 | 44.5 | 104.5 | 0 | 0 |  | 21 | 66.0 | 80.0 | 14.0 | 45.0 | 101.0 | 0 | 1 |
| HbA1c | 33 | 6.1 | 7.8 | 1.7 | 3.5 | 10.4 | 0 | 4 |  | 10 | 6.2 | 7.2 | 1.0 | 4.7 | 8.7 | 0 | 1 |
| Cholesterol | 43 | 4.3 | 5.5 | 1.2 | 2.5 | 7.3 | 0 | 2 |  | 21 | 4.2 | 5.1 | 0.9 | 2.8 | 6.4 | 0 | 3 |
| Triglycerides | 43 | 1.0 | 2.6 | 1.6 | -1.4 | 5.0 | 0 | 0 |  | 21 | 0.9 | 2.2 | 1.3 | -1.1 | 4.2 | 0 | 3 |
| VLDL | 43 | 0.5 | 1.2 | 0.7 | -0.6 | 2.3 | 0 | 0 |  | 18 | 0.4 | 0.8 | 0.4 | -0.2 | 1.4 | 0 | 0 |
| LDL | 43 | 2.6 | 3.6 | 1.0 | 1.1 | 5.1 | 0 | 2 |  | 18 | 2.6 | 3.3 | 0.7 | 1.5 | 4.3 | 0 | 1 |
| LDL | 43 | 1.0 | 1.3 | 0.3 | 0.6 | 1.7 | 0 | 2 |  | 21 | 1.1 | 1.3 | 0.2 | 0.8 | 1.6 | 3 | 0 |
| GGT | 43 | 22.0 | 39.0 | 17.0 | -3.5 | 64.5 | 0 | 6 |  | 21 | 18.0 | 32.0 | 14.0 | -3.0 | 53.0 | 0 | 2 |
| Glucose | 42 | 4.9 | 8.0 | 3.1 | 0.3 | 12.6 | 0 | 6 |  | 21 | 4.9 | 6.2 | 1.3 | 3.0 | 8.1 | 1 | 4 |
| Albumin Creatinine Ratio | 40 | 0.4 | 4.2 | 3.8 | -5.3 | 9.9 | 0 | 5 |  | 21 | 0.4 | 6.4 | 6.0 | -8.6 | 15.4 | 0 | 4 |

*Notes: Lower outliers defined as values that were lower than Quartile 1 (i.e., p25) minus (Interquartile Range (IQR)*1.5. Upper outliers were values greater than Quartile 3 (i.e., p75)+(IQR*1.5). CIND = Cognitive Impairment Not Dementia, BMI = Body Mass Index, VLDL = Very Low Density Lipoprotein, LDL = Low Density Lipoprotein, LDL = High Density Lipoprotein, GGT = Gamma-glutamyl transferase*

**Supplementary Table 3 - Latest recorded risk and protective factors for Cognitive Impairment Not Dementia (CIND) or dementia, among 64 Aboriginal and Torres Strait Islander residents who participated in the Well Person’s Health Check (WPHC) (1998/2000, 2005/2006), by dementia status at follow up (2015-2018)**

| **Risk and protective variables Latest WPHC Measure** | **No - CIND/Dementia (n=43)** | | | **Yes - CIND/Dementia (n=21)** | | | **Tests of significance** | |
| --- | --- | --- | --- | --- | --- | --- | --- | --- |
|  | **N** | **Mean, Med., (%)** | **sd,iqr** | **N** | **Mean, Med., (%)** | **sd,iqr** | **p** | **Effect** |
| Age (Ax.)^a^ | 43 | 63.3 | (9.9) | 21 | 71.0 | (9.9) | 0.005 | -0.775 |
| Age WPHC^a^ | 43 | 50.9 | (10.9) | 21 | 56.9 | (11.7) | 0.049 | -0.536 |
| Sex (Female)^c^ | 29 | (67.4) |  | 16 | (76.2) |  | 0.472 | 0.090 |
| School |  |  |  |  |  |  |  |  |
| Primary^d^ | 11 | (25.6) |  | 9 | (50.0) |  | 0.086 | 0.283 |
| Secondary | 15 | (34.9) |  | 2 | (11.1) |  |  |  |
| Post School | 17 | (39.5) |  | 7 | (38.9) |  |  |  |
| Alcohol drinker | 25 | (58.1) |  | 11 | (52.4) |  | 0.663 | -0.055 |
| Smoker^d^ | 12 | (27.9) |  | 6 | (28.6) |  | 0.956 | 0.007 |
| Physical activity^d^ | 30 | (69.8) |  | 7 | (33.3) |  | 0.008 | -0.346 |
| BMI>=30^c^ | 31 | (72.1) |  | 14 | (66.7) |  | 0.656 | -0.056 |
| HBP^c^ | 19 | (44.2) |  | 12 | (57.1) |  | 0.330 | 0.122 |
| High chol.^c^ | 32 | (74.4) |  | 17 | (81.0) |  | 0.562 | 0.072 |
| Alb.^d^ | 11 | (27.5) |  | 7 | (33.3) |  | 0.769 | 0.061 |
| Diab.^d^ | 21 | (48.8) |  | 9 | (42.9) |  | 0.653 | -0.056 |
| HBG^c^ | 20 | (47.6) |  | 8 | (38.1) |  | 0.473 | -0.090 |
| HBG+Diab.^d^ | 17 | (40.5) |  | 6 | (28.6) |  | 0.355 | -0.117 |
| HBG+Alb.^d^ | 9 | (23.1) |  | 4 | (19.0) |  | 0.718 | -0.047 |
| HBP+Alb.^d^ | 4 | (10.0) |  | 6 | (28.6) |  | 0.063 | 0.238 |
| Diab.+Alb.^d^ | 10 | (25.0) |  | 4 | (19.0) |  | 0.599 | -0.067 |
| Weight (kg)^a^ | 43 | 94.1 | (17.6) | 21 | 82.6 | (12.9) | 0.010 | 0.708 |
| BMI^a^ | 43 | 33.6 | (5.8) | 21 | 31.8 | (4.8) | 0.214 | 0.334 |
| Waist Circ.^a^ | 43 | 111.4 | (14.6) | 21 | 106.4 | (10.4) | 0.167 | 0.372 |
| Vegetables^b^ | 43 | 2.0 | (0.0-4.0) | 21 | 1.0 | (0.0-3.0) | 0.222 | 0.328 |
| Fruit^b^ | 43 | 1.0 | (0.0-2.0) | 21 | 1.0 | (0.0-2.0) | 0.846 | -0.024 |
| Systolic BP^a^ | 43 | 128.9 | (12.7) | 21 | 134.1 | (21.6) | 0.492 | -0.086 |
| Diastolic BP^a^ | 43 | 73.9 | (10.5) | 21 | 74.5 | (11.5) | 0.838 | -0.055 |
| HbA1c^b^ | 33 | 6.6 | (6.1-7.8) | 10 | 6.4 | (6.2-7.2) | 0.634 | 0.073 |
| Cholesterol^b^ | 43 | 4.9 | (4.3-5.5) | 21 | 4.8 | (4.2-5.1) | 0.316 | 0.125 |
| Trigylicerides^b^ | 43 | 1.6 | (1.0-2.6) | 21 | 1.5 | (0.9-2.2) | 0.312 | 0.126 |
| VLDL^b^ | 43 | 0.7 | (0.5-1.2) | 18 | 0.6 | (0.4-0.8) | 0.133 | 0.192 |
| LDL^b^ | 43 | 3.0 | (2.6-3.6) | 18 | 3.0 | (2.6-3.3) | 0.618 | 0.064 |
| HDL^b^ |  | 43 | 1.1 | (1.0-1.3) | 21 | 1.2 | (1.1-1.3) | 0.311 |
| GGT^a^ |  | 43 | 31.0 | (14.1) | 21 | 25.1 | (12.4) | 0.106 |
| Glucose^b^ |  | 42 | 5.4 | (4.9-8.0) | 21 | 5.3 | (4.9-6.2) | 0.303 |
| UACR^b^ |  | 40 | 0.7 | (0.4-4.2) | 21 | 1.3 | (0.4-6.4) | 0.831 |

*Notes: WPHC = Well Person’s Health Check. Latest recorded information was from the second WPHC screen if the person attended, or the first screen if the person did not attend the second screen. Age (Ax.)=Age at dementia screen, Physical activity=Adequate levels (>=20 minutes moderate exercise, >=5 times in the last week), BMI=Body Mass Index, BP=Blood Pressure, HBP=Hypertension, High chol.=High cholesterol, Alb.=Albuminuria HBG=Hyperglycemia, Diab.=Diabetes, Waist Circ.=Waist Circumference, Vegetables=Vegetable Quantity, Fruit =Fruit Quantity, HDL=High Density Lipoprotein, LDL=Low-Density Lipoprotein, GGT=Gamma-Glutamyl Transferase (GGT), VLDL=Very Low-Density Lipoprotein, UACR=Urinary Albumin Creatinine Ratio (UACR)*

*a. Continuous - normally distributed, mean (sd), t-test for differences, Effect Size = Cohen's D*

*b. Continuous - not normally distributed, median (Interquartile Range iqr), ranksum test for differences, Effect Size = Rosenthal (1994) Z score conversion (r = Z/√N)*

*c. Categorical - adequate cell size, n (proportion %), Chi2 test for differences, Effect Size = Cramer’s V*

*d. Categorical – expected cell size <5, n (proportion %), Fisher's Exact test for differences, Effect Size = Cramer’s V*

**Supplementary Table 4 - Univariate and age adjusted risk ratios (RR) from generalized linear model analyses of latest recorded risk and protective factors for Cognitive Impairment Not Dementia (CIND) or dementia, among 64 Aboriginal and Torres Strait Islander residents who participated in the Well Person’s Health Check (WPHC) (1998/2000, 2005/2006), by dementia status at follow up (2015-2018)**

| **Risk and protective variables Latest WPHC Measure** | **Univariate** | | | **Age Adjusted** | | |
| --- | --- | --- | --- | --- | --- | --- |
|  | **RR** | **95%CI** | **p** | **RR** | **95%CI** | **p** |
| Age (Ax.) | 1.05 | (1.01-1.10) | 0.011 |  |  |  |
| Age WPHC | 1.04 | (1.00-1.08) | 0.084 |  |  |  |
| Sex (Female) | 1.35 | (0.57-3.18) | 0.491 | 1.31 | (0.60-2.87) | 0.494 |
| School |  |  |  |  |  |  |
| Primary | Reference |  |  | Reference |  |  |
| Secondary/Post | 0.49 | (0.23-1.04) | 0.064 | 0.58 | (0.27-1.23) | 0.155 |
| Alcohol drinker | 0.86 | (0.42-1.73) | 0.665 | 1.13 | (0.53-2.40) | 0.748 |
| Smoker | 1.02 | (0.47-2.23) | 0.956 | 1.20 | (0.55-2.62) | 0.654 |
| Physical activity | 0.36 | (0.17-0.78) | 0.010 | 0.41 | (0.20-0.85) | 0.016 |
| BMI>=30 | 0.84 | (0.40-1.77) | 0.653 | 0.87 | (0.40-1.86) | 0.713 |
| HBP | 1.42 | (0.69-2.91) | 0.339 | 0.89 | (0.43-1.83) | 0.748 |
| High chol. | 1.30 | (0.51-3.30) | 0.579 | 1.19 | (0.54-2.66) | 0.665 |
| Alb. | 1.19 | (0.58-2.47) | 0.632 | 1.15 | (0.60-2.18) | 0.679 |
| Diab. | 0.85 | (0.42-1.74) | 0.657 | 0.76 | (0.39-1.47) | 0.416 |
| HBG^c^ | 0.97 | (0.86-1.08) | 0.553 | 0.98 | (0.87-1.09) | 0.687 |
| HBG+Diab. | 0.70 | (0.31-1.55) | 0.375 | 0.77 | (0.35-1.70) | 0.521 |
| HBG+Alb. | 0.85 | (0.34-2.11) | 0.727 | 0.95 | (0.40-2.26) | 0.915 |
| HBP+Alb. | 2.04 | (1.05-3.97) | 0.036 | 1.66 | (0.86-3.20) | 0.131 |
| Diab.+Alb. | 0.79 | (0.32-1.98) | 0.615 | 0.91 | (0.38-2.21) | 0.836 |
| Weight (kg) | 0.97 | (0.95-0.99) | 0.002 | 0.97 | (0.94-1.00) | 0.042 |
| BMI | 0.96 | (0.90-1.02) | 0.172 | 0.97 | (0.90-1.05) | 0.429 |
| Waist Circ. | 0.98 | (0.96-1.00) | 0.109 | 0.98 | (0.95-1.01) | 0.110 |
| Vegetables | 0.88 | (0.73-1.07) | 0.192 | 0.91 | (0.76-1.08) | 0.279 |
| Fruit | 1.00 | (0.78-1.29) | 0.982 | 1.03 | (0.81-1.31) | 0.807 |
| Systolic BP | 1.01 | (0.99-1.03) | 0.203 | 1.00 | (0.98-1.02) | 0.893 |
| Diastolic BP | 1.00 | (0.97-1.04) | 0.838 | 1.00 | (0.97-1.03) | 0.931 |
| HbA1c | 0.75 | (0.51-1.08) | 0.122 | 0.79 | (0.49-1.28) | 0.337 |
| Cholesterol | 0.84 | (0.55-1.28) | 0.406 | 0.84 | (0.58-1.23) | 0.374 |
| Trigylicerides | 0.74 | (0.51-1.09) | 0.128 | 0.66 | (0.42-1.03) | 0.067 |
| VLDL | 0.33 | (0.12-0.90) | 0.031 | 0.24 | (0.07-0.79) | 0.019 |
| LDL | 0.78 | (0.46-1.34) | 0.372 | 0.85 | (0.52-1.37) | 0.495 |
| HDL | 2.04 | (0.41-10.01) | 0.381 | 1.62 | (0.29-9.13) | 0.583 |
| GGT | 0.98 | (0.95-1.00) | 0.084 | 0.98 | (0.96-1.01) | 0.260 |
| Glucose | 0.81 | (0.69-0.95) | 0.008 | 0.83 | (0.70-0.97) | 0.021 |
| UACR | 1.00 | (0.90-1.12) | 0.939 | 1.01 | (0.91-1.11) | 0.908 |

*Notes: WPHC = Well Person’s Health Check. Latest recorded information was from the second WPHC screen if the person attended, or the first screen if the person did not attend the second screen. RR = Relative Risk, Age (Ax.)=Age at dementia screen, Physical activity=Adequate levels (>=20 minutes moderate exercise, >=5 times in the last week), BMI=Body Mass Index, BP=Blood Pressure, HBP=Hypertension, High chol.=High cholesterol, Alb.=Albuminuria HBG=Hyperglycemia, Diab.=Diabetes, Waist Circ.=Waist Circumference, Vegetables=Vegetable Quantity, Fruit =Fruit Quantity, HDL=High Density Lipoprotein, LDL=Low-Density Lipoprotein, GGT=Gamma-Glutamyl Transferase (GGT), VLDL=Very Low-Density Lipoprotein, UACR=Urinary Albumin Creatinine Ratio*

**Supplementary Table 5 – Multivariate risk ratios from generalized linear modelling, with bootstrapping variable selection, for earliest and latest recorded risk and protective factors for Cognitive Impairment Not Dementia (CIND) or dementia, among Aboriginal and Torres Strait Islander residents who participated in the Well Person’s Health Check (WPHC) (1998/2000, 2005/2006) and were aged <60 years at baseline measures**

| **Risk and Protective Factors** | **Model 1 (n=44)** | | |  | **Bootstrapping** |  | **Model 2 (n=56)** | | |  | **Bootstrapping** |
| --- | --- | --- | --- | --- | --- | --- | --- | --- | --- | --- | --- |
|  | **RR** | **95%CI** | **p** |  |  |  | **RR** | **95%CI** | **p** |  |  |
| **Earliest WPHC Measures** |  |  |  |  | **(#/200)** |  |  |  |  |  | **(#/200)** |
| **(n=14 CIND/Dementia)** |  |  |  |  |  |  |  |  |  |  |  |
| Age (Ax.) | 1.08 | (1.00-1.16) | 0.050 |  | 154 |  | 1.08 | (1.01-1.15) | 0.016 |  | 138 |
| Primary School | Reference |  |  |  |  |  |  |  |  |  |  |
| Secondary /Post School | 0.42 | (0.07-2.42) | 0.328 |  | 114 |  | 0.38 | (0.17-0.81) | 0.013 |  | 98 |
| Triglycerides | 1.72 | (0.10-30.07) | 0.710 |  | 153 |  | 0.36 | (0.22-0.59) | 0.000 |  | 139 |
| Weight (kg) | 0.96 | (0.91-1.02) | 0.161 |  | 141 |  | 0.96 | (0.93-0.98) | 0.002 |  | 131 |
| Obesity | 0.76 | (0.05-11.07) | 0.842 |  | 30 |  |  |  |  |  |  |
| UACR | 1.01 | (0.93-1.09) | 0.904 |  |  |  |  |  |  |  |  |
| HDL | 4.44 | (0.34-58.87) | 0.258 |  |  |  |  |  |  |  |  |
| Waist Circ. | 0.99 | (0.89-1.11) | 0.883 |  |  |  |  |  |  |  |  |
| VLDL | 0.02 | (0.00-14.39) | 0.241 |  |  |  |  |  |  |  |  |
|  |  |  |  |  |  |  |  |  |  |  |  |
| **Model Information** |  |  |  |  |  |  |  |  |  |  |  |
| Collinearity Index | 56.46 |  |  |  |  |  | 27.85 |  |  |  |  |
| BIC | -119.30 |  |  |  |  |  | -153.12 |  |  |  |  |
